# Characterizing Heated Tobacco Products Marketing on Instagram: Observational Study

**DOI:** 10.2196/43334

**Published:** 2023-03-15

**Authors:** Jiarui Chen, Siyu Xue, Zidian Xie, Dongmei Li

**Affiliations:** 1 Goergen Institute for Data Science University of Rochester Rochester, NY United States; 2 Department of Clinical and Translational Research University of Rochester Medical Center Rochester, NY United States

**Keywords:** IQOS, Instagram, heated tobacco products, web-based tobacco marketing

## Abstract

**Background:**

Heated tobacco products (HTPs), including I Quit Ordinary Smoking (IQOS), are new tobacco products that use an electronic device to heat compressed tobacco leaves to generate an aerosol for consumers to inhale. Marketing of HTPs is prevalent on Instagram, a popular social media platform.

**Objective:**

This study aims to characterize posts related to HTPs on Instagram and their associations with user engagement.

**Methods:**

Through the Instagram application programming interface, 979 Instagram posts were collected using keywords related to HTPs, such as “IQOS” and “heat-not-burn.” Among them, 596 posts were related to IQOS and other HTP marketing. The codebook was developed from a randomly selected 200 posts on the post content by hand coding, which was applied to the remaining 396 Instagram posts. Summary statistics were calculated, and statistical hypothesis testing was conducted to understand the popularity of Instagram posts on HTPs. Negative binomial regression models were applied to identify Instagram post characteristics associated with user engagement (eg, count).

**Results:**

Among Instagram posts related to HTP marketing (N=596), “product display” was dominant (n=550, 92.28%), followed by “brand promotion” (n=41, 6.88%), and “others” (n=5, 0.84%). Among posts within “product display,” “device only” was the most popular (n=338, 61.45%), followed by “heatstick only” (n=80, 14.55%), “accessory” (n=66, 12%), “device and heatstick” (n=56, 10.18%), and “capsule” (n=10, 1.82%). A univariate negative binomial regression model with pairwise comparisons across “product display” types showed that the number of likes for posts with HTP heatsticks was significantly lower compared to posts with HTP devices, accessories, and device-heatstick sets. Multivariate negative binomial regression models showed that HTP-related Instagram posts with a model or lifestyle elements (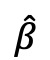;=.60, 95% CI 0.36-0.84) or without obvious product advertising information (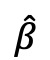
=.69, 95% CI 0.49-0.89) received more likes.

**Conclusions:**

It is shown that posts with product displays were dominant among HTP-related posts on Instagram. Posts with model or lifestyle elements are associated with high user engagement, which might be one of the web-based marketing strategies of HTPs.

## Introduction

Heated tobacco product (HTP) is a tobacco product in which, during its consumption, the tobacco leaves are heated to a point that is sufficient to release nicotine vapor without burning. Produced by Philip Morris International (PMI), I Quit Ordinary Smoking (IQOS) is an HTP with a leading market share [1]. Unlike e-cigarettes that use heated liquid, IQOS uses real tobacco leaves that contain toxins and harmful substances [2]. Globally, there is increasing usage of IQOS. According to PMI’s 2020 third-quarter estimation, the number of total IQOS users increased from 8.8 million to 16.4 million during a 2-year interval [3]. In Japan, only 0.2% of the population used HTPs in 2015, including IQOS and other products. However, in 2019, the proportion increased to 5.8% for IQOS users only [4]. After years of regulatory debate, the US Food and Drug Administration (FDA) approved the sale of IQOS in the US market on April 30, 2019 [5]. Compared to Japan, where IQOS was first launched [6], the United States is still a growing market for IQOS.

On July 7, 2020, the FDA authorized the use of “reduced exposure” (the reduction in the production of harmful and potentially harmful chemicals) for IQOS advertising, which allows IQOS products to be marketed as risk-reduction alternatives to regular tobacco [7]. However, HTPs in general are not risk-free. A previous study showed a similar acute effect of HTPs on cardiovascular health as cigarettes [8]. A recent study showed that there is a potential positive correlation between HTP exposure, including IQOS, and the risk of adverse respiratory symptoms [9]. With the FDA’s “reduced exposure” authorization, IQOS has been aggressively marketed and promoted among nonsmokers and young adults [10,11]. The FDA has been closely monitoring IQOS in the tobacco market in the United States to ensure that the marketing is appropriate and prevents youth access and exposure [3]. Although the import of IQOS has been banned since 2022 due to a patent infringement issue, PMI is fighting to regain the US IQOS market [12].

Social media platforms such as Instagram are now a common platform for the public to share their opinions and are widely used for studying HTPs like IQOS [13,14]. About 71% of the young adults aged between 18 and 29 claimed that they have been Instagram users, which overlaps with the demographic group that IQOS potentially attracts and actual IQOS users [15-18]. A recent investigation on IQOS marketing showed that IQOS has been widely promoted and marketed on Instagram through influential users [14]. One previous study focusing on characterizing the vaping-related content on Instagram through descriptive analyses has yielded informative outcomes, and the success of this vaping-related study indicates that the descriptive analysis on Instagram is a practical method to gain insights into how tobacco products are discussed or even marketed on social media [19].

In this study, the aim is to explore the status quo of HTP-related posts on Instagram by hand coding HTP-related posts and determine possible associations of Instagram HTP-related posts with user engagement by performing statistical analysis. The results of our study provide valuable insights into how HTP is marketed on Instagram, as well as important factors associated with high user engagement on Instagram, which in turn could provide useful information for FDA regulatory science.

## Methods

### Data Collection

Instagram posts related to HTPs were collected through Instagram’s application programming interface, using hashtags related to IQOS and HTPs that were previously collected (heated tobacco, heatnotburn, heatstick, heet, iqo, iqos, iqos3duo, iqosfriends, iqosstore, ploom, and tobaccoheatingsystem) [13]. On June 19, 2021, Instagram posts related to HTPs were collected using a breadth-first search. For each hashtag, a maximum of 5000 Instagram posts were collected. The duplicate posts based on the ID that is unique to each post were removed. Finally, 1000 posts were randomly selected from the remaining posts. Duplicate images from different posts were removed using a Python library named ImageHash and confirmed manually, and 979 posts with distinct images were included in this study [20].

### Hand Coding

The hand-coding process followed an inductive method that was introduced and adopted by previous literature [19,21]. Initially, 200 Instagram posts were randomly selected to develop the codebook through hand coding. After carefully viewing all 200 randomly selected posts, it was first determined if each post was relevant to HTPs, and then all HTP-related posts were grouped based on their content categories (Table 1). The first group contains posts that directly show HTP and HTP-related products (headsticks, devices, flavor capsules, and accessories; Multimedia Appendices 1-3 [22-24]); the second group contains posts on brand promotion (Multimedia Appendix 4 [25]); and the third group contains posts discussing others, such as catch phrases, news, and educational information. In addition, we identified additional content features related to the posts from the first group, “product display,” including model or lifestyle, flavor, product advertisements, and English (Table 2). Each post can have multiple features. The 200 Instagram posts were hand coded by 2 independent human coders in parallel and achieved an agreement rate of 78% (156/200) with a Kappa statistic of 0.73. The discrepancies were resolved by a group of 4 members, including the 2 original human coders. The remaining 779 Instagram posts were then hand coded by the 2 independent coders using the developed codebook. A Kappa agreement of 0.73 was reached on the remaining posts, and the disagreements were resolved using the same procedure as for the sample posts.

**Table 1:** Categories and subcategories of heated tobacco products–related Instagram posts.

Categories and subcategories			Definition
**Product display**			
	Accessory	The post shows accessories for HTP^a^ devices, such as HTP cases, stickers, and so on.	
	Device only	The post shows HTP devices itself.	
	Heatstick only	The post shows either the heatstick or its packages, typically with an indication of the flavor.	
	Both device and heatstick	The post shows both the HTP device and the heatstick.	
	Capsule	The post shows an image of a flavored capsule for HTP.	
**Brand promotion**			
	Brand promotion	The post does not directly show any HTP products. Instead, it shows elements such as a brand logo, retail stores, and so on.	
**Others**			
	Catch phrases	The posts in this category have short pieces of text or phrases as the main body of the images.	
	News	The posts in this category typically show a screenshot of the news articles about HTP.	
	Educational information	The post serves introductory purposes and typically contains basic facts about HTP.	

^a^HTP: heated tobacco product.

**Table 2:** Content features of heated tobacco products–related Instagram posts.

Features	Definition
Model or lifestyle	The image includes figures of models or a suggestion of a lifestyle (eg, a pool, a luxurious car).
Flavor	The image includes information about the flavor of the heated tobacco product.
Product advertisement	The image includes explicit promotional information (eg, discount details, price).
English	Whether the post is in English.

### Statistical Analysis

To examine whether there was a popularity difference among the 3 categories (“product display”, “brand promotion”, and “others”), we calculated summary statistics and conducted statistical hypothesis testing such as 2-sample *t* tests. Similar summary statistics were also applied to the “product display” category to investigate the popularity of different parts of HTPs.

To examine the association of HTP-related Instagram posts with user engagement (the number of likes), the negative binomial regression models were applied in the statistical analysis software R version 4.1.2 (R Core Team). Through pairwise comparisons, the univariate negative binomial regression model was first applied to the “product display” category to examine the difference in the number of likes for the posts with different types of HTPs. The multivariate negative binomial model was then used to identify Instagram post characteristics associated with user engagement, such as product display types and other promotion characteristics including “flavor name mentioned,” “model or lifestyle mentioned,” and so on. Some important account statistics, such as the users’ follower count, the users’ following count, and the total number of media posts by the users, were controlled in our model. The number of followers (the number of Instagram users following this Instagram user), the number of followings (the number of Instagram users that this Instagram user is following), and the number of media (posts) posted by this Instagram user could potentially influence the number of likes of the posts from this user, which should be controlled in the model to eliminate their potential confounding effects.

### Ethics Approval

The study has been reviewed and approved by the Office for Human Subject Protection Research Subjects Review Board at the University of Rochester (study ID: STUDY00006570).

## Results

### Characteristics of Instagram Posts Related to HTPs

Out of 1000 Instagram posts collected using hashtags related to HTPs, we identified 596 Instagram posts related to HTP marketing through hand coding, and 365 of them were IQOS-related. Among all the posts, 550 posts (92.28%) were about “product display,” 41 posts (6.88%) were about “brand promotion,” and the remaining 5 posts (0.84%) were classified as “others” (Table 3). For the “brand promotion” category, approximately half of the posts were about the specific brand IQOS (19/41, 46.34%), and PLOOM-related content made up 12.2% (5/41) of this category. To understand the user engagement of these Instagram posts, we compared the number of likes between different categories. As shown in Table 3, the mean number of likes for posts with “product display” (31.30 likes per post) was higher than that for “brand promotion” (17.21 likes per post) and “others” (29.40 likes per post). The median number of likes for “others” (11 likes per post) was slightly higher than that for “product display” (9 likes per post) and “brand promotion” (9 likes per post). Considering the relatively small sample size for the categories “brand promotion” and “others,” it was decided to combine them as 1 category, “not product display.” Our 2-sample *t* test results showed that the average number of likes for posts in “product display” is significantly higher than that for “not product display” (*P*=.03).

We further divided the 550 “product display” posts into 5 groups based on the product types. Among them, 338 posts (61.45%) were showing “devices only,” 80 posts (14.55%) were showing “heatsticks only,” 66 posts (12%) were showing “accessory,” 56 posts (10.18%) were identified as showing both “devices and heatsticks,” and 10 posts (1.82%) were showing the “capsule” (Table 4). As shown in Table 4, the mean number of likes for accessory-related posts was the highest (38.27 likes per post), while that for the “capsule” category was the lowest (9.90 likes per post). For the median of the like count, “accessory” remained the highest (27.25 likes per post), and heatstick-related posts had the lowest value (10.25 likes per post).

**Table 3:** Number of likes on Instagram posts by category.

Category and subcategory		Number of posts, n (%)	Number of likes, mean (SD)	Number of likes, median (IQR)
Product display		550 (92.28)	31.80 (119.84)	9 (17)
**Not product display**				
	Brand promotion	41 (6.88)	17.21 (22.68)	9 (13)
	Others	5 (0.84)	29.40 (35.97)	11 (19)
	Total	46 (7.72)	18.54 (24.23)	9.5 (14.5)

**Table 4:** Summary statistics of the number of likes for Instagram posts by product display type.

Product display type	Number of posts, n (%)	Number of likes, mean (SD)	Number of likes, median (IQR)
Accessory	66 (12.00)	38.27 (63.83)	16.5 (27.25)
Device only	338 (61.45)	35.47 (147.76)	9 (13)
Device and heatstick	56 (10.18)	29.91 (47.40)	12.5 (26.5)
Heatstick only	80 (14.55)	14.83 (35.98)	3.5 (10.25)
Capsule	10 (1.82)	11.50 (9.90)	9.5 (12.5)

### Instagram Posts Related to HTPs Associated With High User Engagement

**Figure 1:**
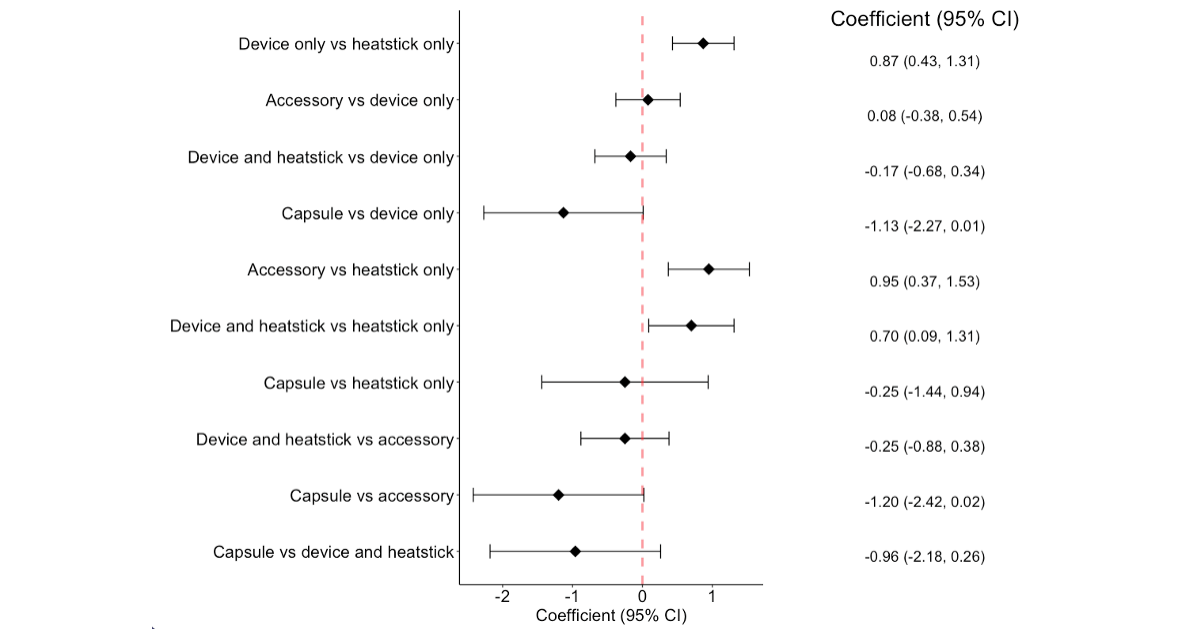
Pairwise comparison of the number of likes among product display types.

As shown by the 95% CI from the negative binomial model in Figure 1, the number of likes for posts within “device only” (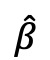
=.87, 95% CI 0.43-1.31), “accessory” (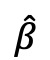
=.95, 95% CI 0.37-1.53), and “device and heatstick” (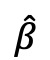
=.70, 95% CI 0.09-1.31) display types were significantly higher than that for posts within “heatstick only” display type.

**Figure 2:**
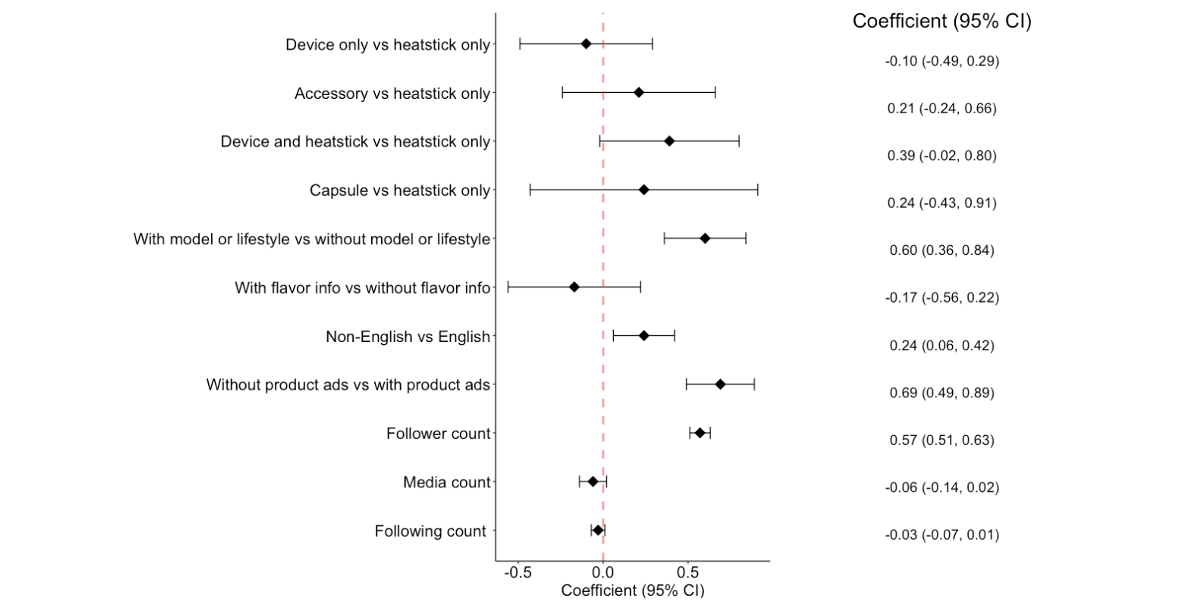
Instagram post characteristics associated with the number of likes.

Our univariate negative binomial regression model on the association of product display type with the number of likes showed major differences between the “heatstick” category and other categories (Figure 1). Thus, the multivariate negative binomial logistic regression model used “heatstick” as the reference category for the product display type variable in the data analysis. As shown in Figure 2, the differences in the number of likes between the “heatstick” category and other categories were not statistically significant after controlling the other variables in the model (95% CI for the coefficients all covered zero). In addition, the models showed that Instagram posts containing images of models or demonstrating pleasant lifestyles received significantly more likes than the posts without these elements, with 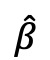
=.60 (95% CI 0.36-0.84). The model also showed that posts without obvious advertising information had a significantly higher number of likes than those with ads (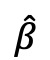
=.69, 95% CI 0.49-0.89). Non-English posts received more likes than English posts (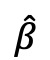
=0.24, 95% CI 0.06-0.42). As we expected, having more account followers significantly leads to a higher number of likes (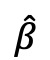
=.57, 95% CI 0.51-0.63).

## Discussion

### Principal Findings

In this study, it is shown that most HTP-related Instagram posts were about “product display.” Among them, posts showing HTP accessories had the highest level of user engagement. Using negative binomial regression models, it is observed that, among the 5 categories of product display, posts showing only the heatstick received significantly fewer likes than the ones showing HTP devices, accessories, or the HTP set containing both the device and heatstick. It also showed that the presence of models, lifestyle, or the absence of advertising information might be contributing factors to the high number of “likes” on HTP-related Instagram posts.

### Comparison With Previous Studies

This study showed that the inclusion of model or lifestyle elements could improve user engagement. As reported before, a common strategy for IQOS marketing is to associate the products with elements such as coffee and food [2]. These elements fall into the lifestyle feature using our classification method. In addition, another study stated that sex appeal is an important element in IQOS marketing [26], which corresponded to the model feature in our study. Therefore, HTP-related Instagram posts, including images of models, attracted more social media users and therefore became a popular marketing strategy.

Among HTP-related Instagram posts in our study, posts with product displays were dominant. One previous study on e-cigarettes showed that 81.5% of the provaping posts on Instagram were about product display [19]. Furthermore, the number of provaping posts on Instagram significantly outweighs the number of antivaping posts [19]. Therefore, similar results from both e-cigarettes and HTPs suggest that “product display” might be one of the most prevalent types of posts for tobacco products on Instagram.

The results from negative binomial regression models suggest that posts without advertisement information received more likes, indicating high user engagement. A previous study on the advertisement on social media showed that only 13.02% of users found the advertisement on Instagram informative and useful, a percentage significantly lower when compared to Google (33.53%), Pinterest (29.72%), and Twitter (21.21%) [27]. The low user engagement of Instagram posts with advertisements might result from psychological reactance (an urge to resist the initial intention of the message) [28,29].

### Limitations

This study has several limitations. In terms of the study methodology, during data collection, the set of hashtags that were used may not have been comprehensive enough to capture all the related posts on Instagram. Therefore, our results may not be representative. Additionally, the data set may include boosted posts, which are paid posts that receive more exposure and are thus more likely to receive more likes. With the data we collected, it is hard to distinguish the boosted posts, which could potentially introduce some bias into the results. Moreover, even though the agreement rate between 2 human coders for hand coding Instagram posts was high, this process might introduce some biases. Lastly, only the posts from a specific time interval were collected. However, HTP-related Instagram posts and the user engagement of these posts might evolve. Therefore, a longitudinal study might be necessary in the future.

In terms of result interpretation, as of February 2021, 71% of adults between 18 and 29 in the United States claimed that they ever used Instagram, while the percentage was only 48% for people between 30 and 49 and 29% for people between 50 and 64 [30]. Therefore, the findings might not represent the whole population. In addition, this collected data revealed disproportionally more “product display” posts than other categories, which may potentially introduce some bias into our results.

### Conclusions

This study showed the relatively high popularity of HTP-related Instagram posts that display product images. More specifically, the posts containing images of HTP accessories had the highest user engagement level. Considering the underage Instagram users, these Instagram posts with HTPs should be closely monitored and potentially regulated to prevent youth and young adults from using these tobacco products. In addition, it showed that the user engagement level of Instagram posts with the presence of a model or lifestyle, or the lack of advertising elements, was high. These findings could help us design anti-HTP Instagram posts with high user engagement to educate the public about the risk of HTP use in the future. Future studies could take demographic or geographic factors into account to determine how different demographic groups react differently to these HTP-related posts on Instagram.

## Appendix

Multimedia Appendix 1 Example image for the “device only” category, reproduced from Marta [22], which is published under Creative Commons Attribution 4.0 International License [31].

Multimedia Appendix 2 Example image for the “heatstick only” category, reproduced from Lee [23], which is published under Creative Commons Attribution 4.0 International License [32].

Multimedia Appendix 3 Example image for the “device and heatstick” category, reproduced from ACBSA [24], which is published under Creative Commons Attribution 4.0 International License [31].

Multimedia Appendix 4 Example image for the “brand promotion” category, reproduced from Tokumeigakarinoaoshima [25], which is published under Creative Commons Attribution 4.0 International License [31].

## Abbreviations

FDA: Food and Drug Administration
HTP: heated tobacco product
IQOS: I Quit Ordinary Smoking
PMI: Philip Morris International

## Data Availability

The data are available upon reasonable request from the corresponding author.
