# Supplementary figures and images for: Characterizing Heated Tobacco Products Marketing on Instagram: Observational Study
Source: JMIR Form Res. 2023 Mar 15;7:e43334. doi: 10.2196/43334 (PMC10131776; doi:10.2196/43334)

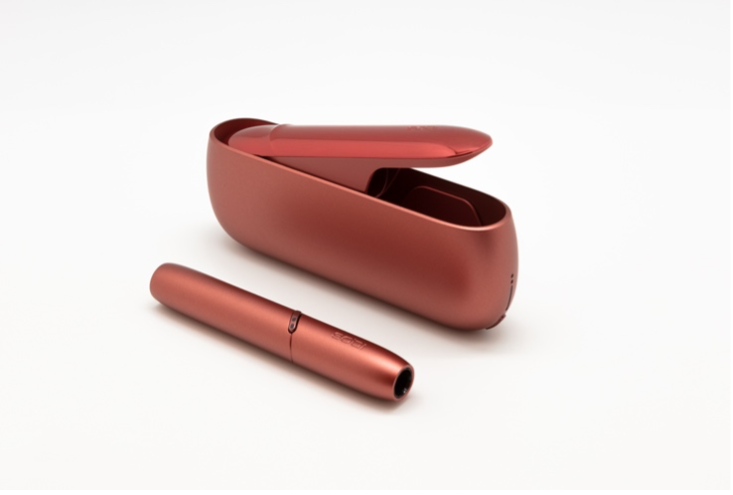

Supplement: Multimedia Appendix 1 [file formative_v7i1e43334_app1.png]

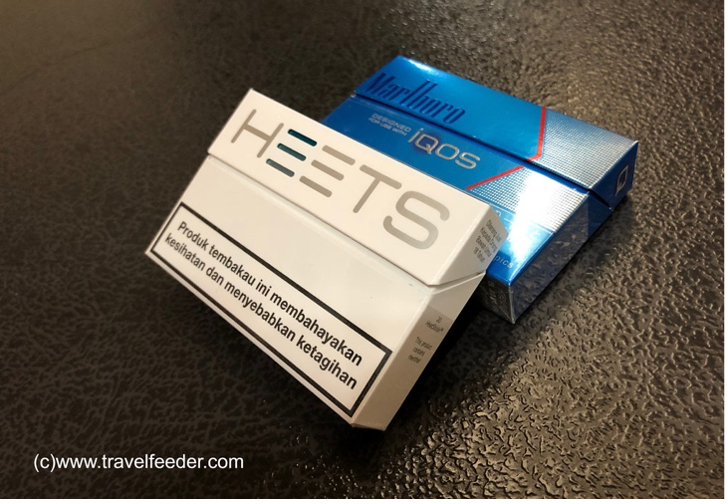

Supplement: Multimedia Appendix 2 [file formative_v7i1e43334_app2.png]

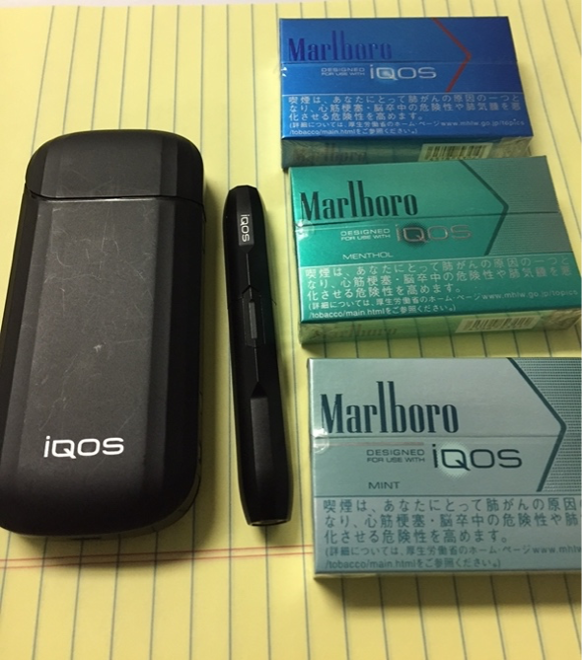

Supplement: Multimedia Appendix 3 [file formative_v7i1e43334_app3.png]

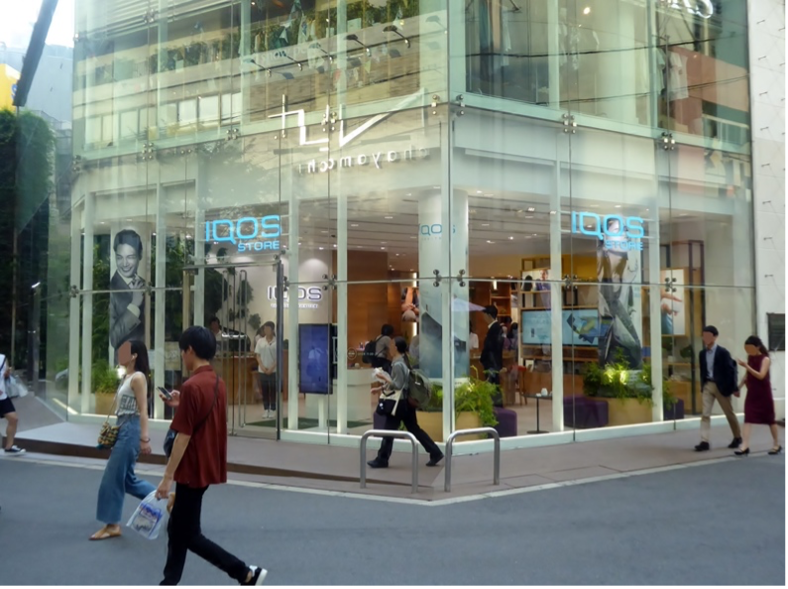

Supplement: Multimedia Appendix 4 [file formative_v7i1e43334_app4.png]
